# Obstacle Detection System for Navigation Assistance of Visually Impaired People Based on Deep Learning Techniques

**DOI:** 10.3390/s23115262

**Published:** 2023-06-01

**Authors:** Yahia Said, Mohamed Atri, Marwan Ali Albahar, Ahmed Ben Atitallah, Yazan Ahmad Alsariera

**Affiliations:** 1 Remote Sensing Unit, College of Engineering, Northern Border University, Arar 91431, Saudi Arabia; 2 King Salman Center for Disability Research, Riyadh 11614, Saudi Arabia; 3 Laboratory of Electronics and Microelectronics (LR99ES30), University of Monastir, Monatir 5019, Tunisia; 4 College of Computer Sciences, King Khalid University, Abha 62529, Saudi Arabia; matri@kku.edu.sa; 5 School of Computer Science, Umm Al-Qura University, Mecca 24382, Saudi Arabia; mabahar@uqu.edu.sa; 6 Department of Electrical Engineering, College of Engineering, Jouf University, Sakaka 72388, Saudi Arabia; abenatitallah@ju.edu.sa; 7 College of Science, Northern Border University, Arar 91431, Saudi Arabia; yazan.sadeq@nbu.edu.sa

**Keywords:** visually impaired people, deep learning, obstacle detection, object detection, neural architecture search (NAS), anchor-free model

## Abstract

Visually impaired people seek social integration, yet their mobility is restricted. They need a personal navigation system that can provide privacy and increase their confidence for better life quality. In this paper, based on deep learning and neural architecture search (NAS), we propose an intelligent navigation assistance system for visually impaired people. The deep learning model has achieved significant success through well-designed architecture. Subsequently, NAS has proved to be a promising technique for automatically searching for the optimal architecture and reducing human efforts for architecture design. However, this new technique requires extensive computation, limiting its wide use. Due to its high computation requirement, NAS has been less investigated for computer vision tasks, especially object detection. Therefore, we propose a fast NAS to search for an object detection framework by considering efficiency. The NAS will be used to explore the feature pyramid network and the prediction stage for an anchor-free object detection model. The proposed NAS is based on a tailored reinforcement learning technique. The searched model was evaluated on a combination of the Coco dataset and the Indoor Object Detection and Recognition (IODR) dataset. The resulting model outperformed the original model by 2.6% in average precision (AP) with acceptable computation complexity. The achieved results proved the efficiency of the proposed NAS for custom object detection.

## 1. Introduction

The primary sensory organ of a person is his or her eyes. In our environment, visual information provides help to perform any task. However, this fact presents a substantial challenge that the visually impaired encountered in their lives and can isolate them from society. For this reason, it is common for sighted individuals (e.g., friends or family) to be overly excited to help visually impaired people, but very frequently, rushing to help them without asking can cause visually impaired people to feel helpless and compromise their independence. This possibility implies a negative impact on their confidence and their emotional well-being. Consequently, it is essential to allow the visually impaired to support themselves, helping them to increase their autonomy and integration into society. However, the lack of accessibility to information by visually impaired people limits their independence. Another challenge for the visually impaired, especially those with complete vision loss, is navigating around places. The visually impaired must avoid obstacles to ensure their safety. As a solution, an artificial vision system can be designed to detect obstacles and warn the visually impaired person to avoid obstacles. In effect, an object detection system must be used to detect obstacles for further processing.

Object detection is one of the most studied tasks in computer vision and has been extensively investigated. Recently, most object detection methods have relied on convolutional neural networks (CNNs) [1]. CNNs have been widely deployed for different computer vision tasks, such as image classification [2], object detection [3], scene recognition [4], indoor object detection [5], and many others [6]. High performances have been achieved by state-of-the-art object detection frameworks, such as faster region-based CNNs (R-CNNs) [7], You Only Look Once (Yolo) [8], single shot multi-box detection (SSD) [9], and EfficientDet [10]. However, all object detection frameworks have a complicated design compared to those used for classification due to the need to localize and classify multiple objects simultaneously on an image. In contrast, classification requires predicting the label of a single object on the image. Due to the presented complexity of the object detection task, designing a high-performance model requires substantial manual effort to find the perfect hyperparameters.

Neural architecture search (NAS) [11] has recently proved its performance for automatically searching high-performance neural network models in large-scale search spaces. NAS are data-driven, unlike manual, experience-driven methods requiring less expert intervention. The NAS workflow can be presented in three main stages. It first defines the search strategy and sampling candidate architecture from a given search space. Second, it evaluates the performance of the candidate architecture. Finally, it updates the configuration and parameters based on the achieved performances.

The main limitation of deploying NAS for a wide range of applications is the need for extensive computation overhead. In particular, the evaluation process is computationally extensive and time-consuming because it comprises full training and validation processes. To overcome this issue, a proxy task is applied to reduce the evaluation procedure time. Practically, the proxy task requires scaling down the training iterations, network parameters, and input data. However, the performance of the proxy task and the desired task presents a wide gap, and the evaluation process is biased. Therefore, designing a proxy task with high performance and efficiency for a particular task is challenging and requires high effort. Another solution to avoid high computation requirements by the NAS method is to build a supernet that covers the entire search space and then train candidate architectures based on a shared parameters method [12]. However, this method requires substantial memory and reduces the size of the search space.

To our knowledge, deploying NAS methods for object detection networks has rarely been investigated. Most methods focus on the classification task and apply transfer learning to move to the detection task. Devising an accurate and efficient NAS method for object detection tasks is essential but presents many challenges. To this end, a new NAS method is proposed in this work to search for an object detection network. The proposed NAS method was based on reinforcement learning [13] with an LSTM-based controller. The proposed method is computationally fast, requires less memory, and finds high-performance models while saving search time.

To achieve real-time processing of our obstacle detection system, we proposed using the Fully Convolutional One-Stage detector (FCOS) [14] as a baseline model. We also applied the proposed NAS to search the Feature Pyramid Network (FPN) architecture and the output prediction head. The FCOS model is an anchor-free object detection model with many advantages compared to other one-stage detectors. For example, detecting objects without predefined anchors reduces the number of hyperparameters, decrease the training time and simplifying it. In addition, the anchor-free model eliminates complex computations, such as anchor matching and intersection over union (IoU) calculation, which accelerates the training testing and inference while reducing memory occupation.

Moreover, we proposed using the efficientNet [15] as a backbone guaranteeing that the resulting model fits into embedded devices. The efficientNet is a CNN model designed for implementation on mobile devices with a lightweight size and high performance. Additional compression techniques have been applied to the model to reduce its size and accelerate its inference speed. A channel pruning technique [16] was applied to filter channels from redundant and weak ones. This technique eliminates unnecessary channels to reduce the model size and accelerates inference time. A post-training quantization technique [17] was applied by replacing a 32-bit floating-point representation with a 4-bit fixed-point representation for both activations and weights.

A set of objects were collected from two datasets to train and evaluate the proposed obstacle detection model. First, to collect the outdoor obstacle, we searched the Coco dataset [18] for possible target objects that could be considered obstacles for visually impaired people. Second, the Indoor Object Detection and Recognition (IODR) dataset [19] was used for indoor obstacles. These datasets were combined to create an indoor and outdoor obstacle detection dataset. For the remaining classes in the Coco dataset, we considered them negative samples.

The main contributions of this work are the following:

- Proposing an obstacle detection system for navigation assistance of visually impaired people;
- Collecting a dataset of indoor and outdoor obstacles to train and evaluate the proposed system;
- Proposing a NAS method to search the FPN and the prediction head of an object detection model by carefully specifying the search space design, the proxy task, and the evaluation strategies;
- Proposing the use of an anchor-free object detection framework to achieve real-time processing and reduce the memory footprint;
- Employing an efficient backbone model to guarantee embedded implementation;
- Compressing the resulting model to fit into embedded devices; and
- Proving the efficiency of the proposed NAS method by achieving high performance compared to the original FCOS framework.

The remainder of this paper is organized as follows: Section 2 is reserved for discussing related works. In Section 3, we explain and detail the proposed approach. The experiment and results are presented and discussed in Section 4. Finally, in Section 5, we provide conclusions and discuss future works.

## 2. Related Works

Detecting obstacles for assisting visually impaired people in their navigation has been rarely investigated, and few works exist [5,6,20]. However, object detection has a wide diversity, and many studies have been performed. This section discusses the most recent object detection frameworks, focusing on those designed through NAS.

In the literature, object detection frameworks can be divided into two main categories: those with one stage and those with two stages. The two-stage detection framework comprises a region proposal mechanism and a detection network. The region proposal convolutional neural networks (R-CNN) [21] family is the most dominant, and all recent works have been based on its concept. This framework is based on an external region proposal mechanism, a convolutional neural network for feature extraction, and a support vector machine for classification. It presents an extensive computation overhead and a complicated training process. It was followed by the faster R-CNN [7] framework, which eliminates the external region proposal network and replaces it with an integrated, fully convolutional neural network called a region proposal network (RPN). The new version has achieved higher detection accuracy but is still computationally extensive with a slow processing speed.

The one-stage detection framework has a simple architecture compared to two-stage detectors. This category directly predicts the localization bounding box and object class for each location on the output feature maps generated by the backbone. Therefore, one-stage detection frameworks are much faster than two-stage frameworks, with comparable accuracies. Yolo [8] is one of the best one-stage detection frameworks. It solves the detection task as a regression problem by predicting the localization bounding box and associating a confidence score with each class label. Yolo is fast, making it suitable for real-time processing, but it struggles to detect small and close objects. The third version of Yolo [22] was proposed to solve this problem by introducing the detection of objects at different scales and merging the detection for the final output.

The main common point between the two categories is prediction based on testing anchors at different scales and aspect ratios. Unfortunately, using anchor boxes increases the number of hyper-parameters, which requires more computation resources and longer training times.

Recently, a new type of one-stage detection framework [23,24] was proposed. This type has eliminated the use of an anchor box, expediting the processing time and reducing the computation complexity and the required resources. cornerNet [24] is one of the anchor-free object detection frameworks. The main idea of cornerNet is to detect objects with two points: the top-left corner of the bounding box and the bottom-right corner based on a CNN. This technique allows for eliminating the need for predefined anchors. In addition, a corner pooling layer was proposed to improve network capability in detecting corners to enhance performance. As a result, cornerNet has achieved state-of-the-art performance with 42% AP on the Coco dataset [16] and with lower computation overhead.

CenterNet [25] has proposed detecting objects by their center points and corner points. This framework was based on cornerNet with enhancement in precision. Additional custom layers were added to collect rich features on the top-left and bottom-right corner points and the center point of the cropped region. The proposed cascaded corner pooling and center pooling layers effectively achieved better detection performances. As a result, the framework achieved the top AP of 47% on the Coco dataset [16] while being faster.

As discussed above, object detection frameworks are very complicated and require highly qualified experts to design an effective framework that achieves the desired performance. However, the appearance of the NAS method has allowed for searching for new architectures in a vast research space that is most suitable for each task. On the other hand, the NAS method is computationally extensive and time-consuming, limiting its use for many applications. Traditional NAS methods require up to 15,000 GPU-days [26] to find a suitable architecture. Recent works have reduced the search time to 0.2 GPU-days [27] by building a supernet that considers the complete search space and performing a single training for all candidates based on weight sharing and bi-level optimization [28]. This technique reduces the search time but requires substantial memory. Therefore, this NAS method cannot be applied to searching complex neural networks.

To avoid the aforementioned problem, a single-path training approach [29] was proposed to reduce bias caused by model simplification and approximation of the designed supernet. DetNAS [30] deployed the single path training approach to search for a high-performance object detection framework. However, this approach presented one limitation: restricting the search space to a sequential structure. As a result, the single path sampling of the weight gradients has introduced large variance into the optimization process. Such variance limits the search for complex architectures and restricts the NAS method to apply only simple variations, such as modifying the kernel size of predefined blocks.

Object detection frameworks are highly complicated and differ vastly from image classification models, especially at the feature merging level and in their output predictions. The feature pyramid network (FPN) [31] was proposed to handle feature merging and parallel prediction in the object detection framework. Due to its importance for object detection, NAS-FPN [32] was proposed to search for the best FPN architecture for the RetinaNet object detection framework [33]. The FPN architectures were modeled with a recurrent neural network controller, trained using reinforcement learning. The proposed method is time-consuming even with a proxy task based on the ResNet [34]. A comparison of different object detection models based on various criteria is provided in Table 1.

After a deep study of the literature, we discovered that all existing NAS methods for object detection are restricted to the backbone design, or they target the FPN architecture. In this work, we designed a NAS method to search for the FPN architecture and the prediction head. To reduce the computation overhead, we applied the proposed NAS on an anchor-free object detection framework, FCOS [14], which allows for reducing the search time by eliminating anchor matching. Compared to an anchor-based framework, FCOS is significantly faster with higher performance. The following section provides a detailed description of the proposed approach.

## 3. Proposed Approach

In this work, we proposed a fast NAS method to search for the FPN and the prediction head of an anchor-free object detection framework. The used framework facilitated the application of the NAS method and reduced the search time.

### 3.1. Problem Formulation

The proposed search method was based on FCOS, a simple and effective object detection framework. As input (X), it takes an image with a size of h × w × 3, where 3 refers to the number of channels of the RGB color space, h is the height of the image, and w is the width of the image. As output (Y), a list of tensors $y_{s}$ is generated, and the size of each tensor is ((k + 4 + 1) × $h_{s}$ × $w_{s}$), where ($h_{s}$ × $w_{s}$) is the dimension of the feature map at level p of the FPN, and (k + 4 + 1) is the number of output channels. K refers to the number of detection classes, 4 is the number of parameters of the detection bounding box, and 1 is the centerness factor.

Therefore, the FCOS takes an input X and predicts an output Y. The framework has three main components: the backbone, FPN, and prediction head. In the first stage, features were extracted by mapping the input X to feature maps at different levels C = {$c_{3}$, $c_{4}$, $c_{5}$} with a resolution of ($h_{i}\times w_{i}$) = h/$2^{i}$ × w/$2^{i}$. In the second stage, the FPN maps the feature maps c to pyramid feature maps P = {$p_{3}$, $p_{4}$, $p_{5}$, $p_{6}$, $p_{7}$}. In the final stage, the prediction head generates the final prediction Y by collecting information from different levels of the pyramid features.

Detecting objects at different scales and aspect ratios requires various receptive fields. Therefore, selecting and merging feature maps from different levels require an effective mechanism. Due to the high importance of this mechanism for object detection tasks, most researchers have focused on the design of the FPN and multi-level prediction head while deploying existing efficient backbones. Considering this concept, the main goal of this work is to search for an effective FPN to decide how and when to merge the feature maps and a prediction head to detect objects at different scales and aspect ratios while adopting an existing backbone to ensure lightweight size and embedded implementation.

The original FCOS with the proposed backbone was pretrained on the target dataset to improve the framework’s performance. Then, the backbone parameters were fixed, and the search process was started. The proposed NAS method searched the FPN and the prediction head as the main components of the object detection framework. The FPN extracted features from different pyramid levels. The prediction head collected information from the feature maps in p and merged them to generate the final prediction to avoid the overfitting problem. The main discussion concerns diversifying the FPN to detect relevant features and the number of layers in the prediction that merged the collected information from different levels. In this work, we deployed the NAS method to search for the best combination by automatically testing all possibilities.

### 3.2. Search Space

In this work, we search for different components of an object detection framework. Since there is a difference between the FPN and the prediction head, two separate search spaces were considered. For the FPN architecture, a basic block was designed with modified connections. For the prediction head, a sequential search space was considered. For more flexibility, the cell structure was replaced by atomic operations. To create a basic block, two layers were selected from the sampling pool L with ID 1 and ID2, operations op1 and op2 were applied to each layer, and a final aggregation operation was applied to fuse the output into a single feature. Multiple basic blocks were stocked to create a deep structure, and those blocks’ outputs were added to the sampling pool. At step time t, the basic block $b_{t}$ transforms the sampling pool $L_{t-1}$ to $L_{t}$ = $L_{t-1}\cup \{l_{t}\}$ where $l_{t}$ is the output of the basic block $b_{t}$.

Only depthwise convolution layers were considered to design a more efficient decoder. Additionally, to enable the decoder to perform convolution filters on irregular feature maps, we considered the 3 × 3 deformable convolution layers. For the aggregation operation, element-wise addition and concatenation, followed by a 1 × 1 convolution layer, were considered. Table 2 presents the considered operations in the proposed search process.

**Figure 1:**
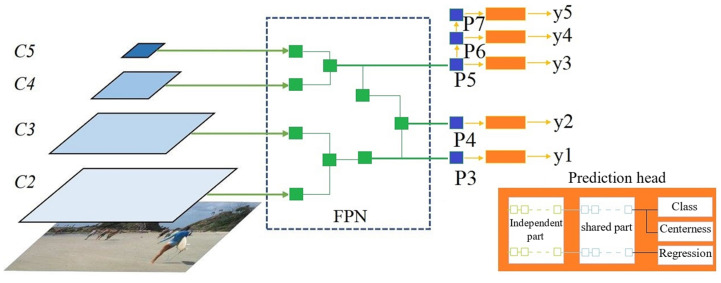
The concept of the proposed architecture (the numbers of layers and internal connections are presented for illustration purposes and do not represent the real ones).

The proposed decoder has three main components: FPN, prediction head, and weight sharing. The concept of the proposed architecture is presented in Figure 1.

As discussed earlier, the FPN mapped feature maps C to feature maps P. We started by setting the sapling pool $L_{0}$ = C. Then, the FPN architecture was defined by applying seven consecutive basic blocks. To produce the pyramid structure of feature maps P, the output of the last three basic blocks was collected, where {$x_{5}$, $x_{6}$, $x_{7}$} were mapped to {$p_{3}$, $p_{4}$, $p_{5}$}.

To create a shared scheme across layers, a combination rule was proposed. If any intermediate layer $x_{t}$ was not sampled with the previous basic block and did not belong to the last three layers, element-wise addition was performed to merge this layer to generate the output features. The proposed rule can be computed using Equation (1). Similar to the aggregation function, if the feature maps presented different dimensions, the bilinear interpolation was used for upsampling the smallest one. For $p_{6}$ and $p_{7}$, they were obtained in the same way with FCOS by applying a 3 × 3 convolution layer with a stride of 2 on $p_{5}$ and $p_{6}$ respectively.

(1) $$p_{i}^{*}=p_{i}+x_{t}$$

The prediction head provides predictions of the class and localization bounding box by collecting information from the pyramid architecture of the feature maps P. In the original FCOS, the prediction head comprised four 3 × 3 convolution layers. A sequence of six layers defined the prediction head. The basic operations of the prediction head were different from those of the FPN. Additional convolution layers were added, where regular 3 × 3 convolution layers and 1 × 1 convolution layers were added to the sampling pool. In addition, the same as FCOS, the batch normalization layer was replaced by the group normalization layer [34], allowing for weight sharing between different levels.

To make the weight-sharing technique at the prediction head more flexible, we introduced an index *i* to manage the weight-sharing start point. For all layers, before the index, *i* weights were processed independently. Otherwise, global weights were shared. By considering the independent part of the prediction head as an extension to the FPN and the shared part as a variable-length prediction head, it is possible to manage the computations of the individual FPN branch. Furthermore, such a configuration allows for extracting specific features at different levels and generating predictions by sharing features across all levels.

### 3.3. NAS Method

**Figure 2:**
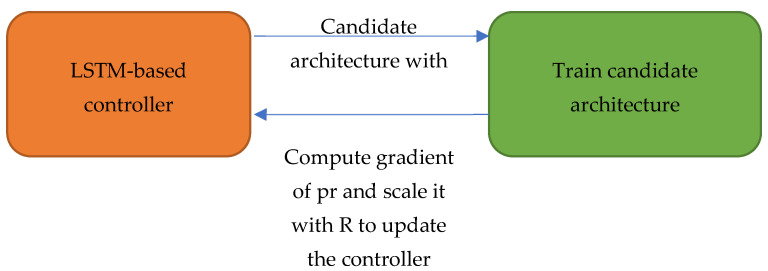
Workflow of the proposed NAS method.

The proposed NAS method was based on reinforcement learning with an LSTM-based controller. The main idea was to specify hyperparameters and connection configuration using a string former, such as filter width: 3, filter height: 3, num filter: 32, and so on. The LSTM-based controller generated those strings for building the network architecture. Then, the candidate architecture was trained with the target dataset and evaluated using the validation data. Based on the achieved reward, a reinforcement learning algorithm was used to update parameters in the controller. The workflow of the proposed NAS method is presented in Figure 2.

Reinforcement learning was used to train the controller. The reward was considered to discover the top-performance candidate architecture by forcing the controller to maximize the expected reward. The update of the controller can be computed as Equation (2):

(2) $$J\left(\theta_{c}\right)=E_{p(a_{1:T;\theta_{c})}}(R)$$

The reward value is non-differentiable, imposing the use of the policy gradient to update the controller parameters iteratively. First, the reinforcing method was applied to achieve the update. Subsequently, an empirical approximation, normalized by the number of candidate architecture m generated in one batch, was performed to make the update smoother. The number of hyperparameters to be predicted for the architecture by the controller was limited to T. The update can be computed as Equation (3):

(3) $$J\left(\theta_{c}\right)=\sum_{t=1}^{T}E_{p(a_{1:T;\theta_{c})}}\left({\nabla \theta }_{c}\log pr\left(a_{t}|a_{(t-1):1};\theta_{c}\right)R\right)\;J\left(\theta_{c}\right)=\frac{1}{m}\sum_{k=1}^{m}\sum_{t=1}^{T}\left({\nabla \theta }_{c}\log pr\left(a_{t}|a_{\left(t-1\right):1};\theta_{c}\right)R_{k}\right)$$

The proposed update presents high variance with an unbiased gradient estimation. A bias function bf was adopted to minimize the variance of this estimation. If this function is independent of the current action, then the gradient estimation is unbiased. Therefore, the exponential moving average of the previous reward was employed as a bias function. The final update can be computed as Equation (4):

(4) $$J\left(\theta_{c}\right)=\frac{1}{m}\sum_{k=1}^{m}\sum_{t=1}^{T}\left({\nabla \theta }_{c}\log pr\left(a_{t}|a_{\left(t-1\right):1};\theta_{c}\right){(R}_{k}-bf)\right)$$

In this work, a progressive search strategy was deployed instead of a joint search strategy for both the FPN and the prediction head because FPN requires less computation and a shorter search time than the prediction head. The training data were randomly divided into meta-train (MT) and meta-validation (MV) subsets. The backbone was pre-trained and fixed to accelerate the training process, and the output feature map C was stored in the cache. This strategy makes the training time independent of the complexity of the backbone model. It was proved that backbone fine tuning could be eliminated if the pre-trained feature maps were powerful enough to cause the framework to converge. Additionally, acceleration techniques, such as Polyak weight averaging, were applied to expedite the training process [35].

In object detection, average precision (AP) is the most used metric. Object detection is a complicated task in which proposed models achieve very low AP at the early training stage. Therefore, the model cannot provide information about network architecture (a) performance, making the search process time-consuming and computationally extensive. The negative loss sum was considered instead of the AP as the reward for facilitating architecture evaluation at the early stages. The proposed reward can be computed as Equation (5):

(5) $$R\left(a\right)=\sum_{(x,Y)\in MV}(L_{cls}(x,Y|a)+L_{reg}(x,Y|a)+L_{ctr}(x,Y|a)$$

where $L_{cls}$ is the class loss, $L_{reg}$ is the regression loss, and $L_{ctr}$ is the centerness loss.

The gradient of the LSTM-based controller was estimated using the proximal policy optimization (PPO) [36].

## 4. Experiment and Results

### 4.1. Implementation Details

#### 4.1.1. Search Process

A fast proxy task was proposed to search for the best architecture for the model decoder, composed of the FPN and the prediction head. This proxy task allows for fast evaluation of the sampled architecture. The MS Coco dataset was used as the proxy dataset. The dataset contains 118 K images for training and 41 K images for testing and provides bounding box annotations of 80 classes. The transfer learning technique was applied in the training phase because different datasets will be used for the real task. The data were already divided into meta-training and meta-validation sets. Each architecture sampled by the controller was trained on the mat-train data, and the reward was computed on the mat-validation data (testing set).

The input data size was set to 384 × 384, and the size of the target objects was scaled correspondingly. The Adam optimizer was used for training with an initial learning rate of 0.0008 and a batch size of 128. A decay rate of 0.9 was set for the Polyak averaging technique. After every 300 iterations, the evaluation process was performed. For a fast search, the output feature maps of the backbone were stored in cache memory for prompt communication. To enhance the quality of the features generated by the backbone, they were initialized by publicly available pre-trained weights. Then, they fine-tuned those weights on the MS Coco dataset based on the FCOS strategy. It is worth mentioning that the fine-tuning process was performed only at the start of the search stage.

To search for the FPN and prediction head, a progressive strategy was deployed. First, the FPN was explored, and the original FCOS prediction head was used. The majority of operations in the FPN architecture have 64 output channels. Therefore, the inputs of the FPN were resized for compatibility with its output channels width, using a 1 × 1 convolution layer. After obtaining the best FPN structure, the prediction head was searched. To do so, the same parameters used for searching the FPN were adopted with minor modifications. Considering that the prediction head requires more features, the output channel width was doubled from 64 to 128.

**Figure 3:**
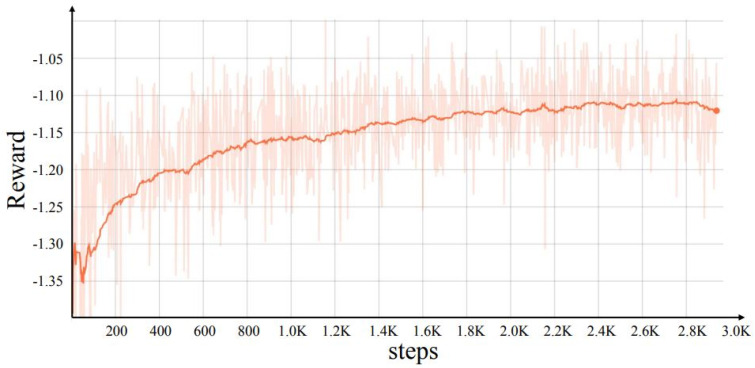
Reward performances in the proxy task. Reward increases indicate that the training reinforcement learning model is working.

For searching the FPN, the model of the controller converged, searching more than 2.8 K structures based on the proposed proxy task. The reward of the controller model is presented in Figure 3. Subsequently, the best top-20 structures were selected for the full training process. The best performing FPN structure from the top 20 was selected to search the prediction head, and the search process was performed. The model of the controller converged after only 600 rounds, which is very fast compared to searching the FPN. Then, the top 10 best performance prediction heads were selected for the full training process. The search process of both the FPN and the prediction head lasted 4 days on a cloud-based 8 Nvidia V100 GPU.

#### 4.1.2. Full Training Process

In the full training process, the searched models were trained on the proposed combination of the MS Coco and IODR datasets. Then, the models were evaluated using the testing set, and the best model was selected. The primary purpose of this work is to build an obstacle-detection system for assisting visually impaired people in their navigation. Therefore, only objects that could be considered obstacles were considered from both datasets. The considered classes are presented in Table 3. The same configuration was applied for a fair comprising of the original FCOS by resizing the images to a minimum size of 800 pixels and a maximum size of 1333 pixels. The training process was performed using a cloud-based 4 Nvidia V100 GPU. Each model was trained for 90 K iterations with a batch size of 16. The learning rate was initialized to 0.01 and devised by 10 in the 60 K-th iteration and at the 80 K-th iteration. Model improvement techniques were only applied to the best-performing model.

### 4.2. Search Results

**Figure 4:**
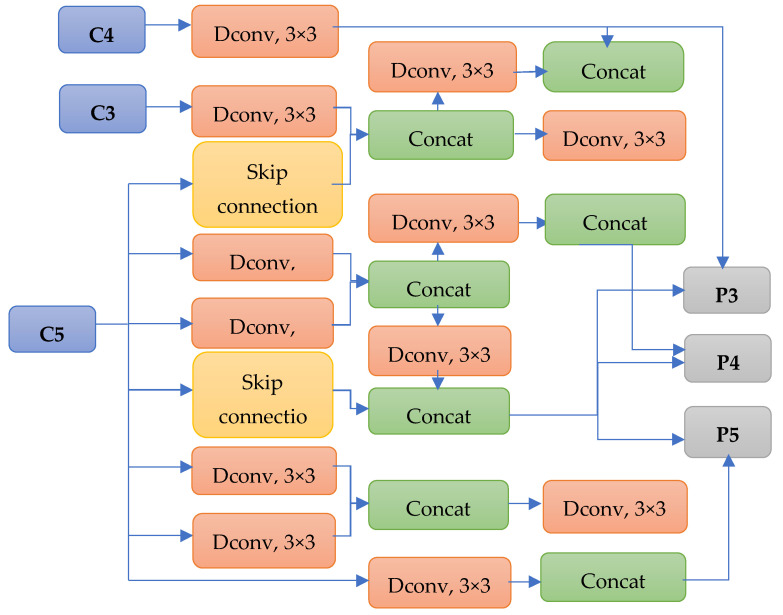
Best performance searched FPN (Dconv refers to deformable convolution).

After training and evaluating the models, we discovered the best FPN and prediction head structure. The FPN structure with the best performance is presented in Figure 4. The controller removed connections with C2 features and achieved the best performance. In addition, the controller discovered that deformable convolution layers with a 3 × 3 kernel and skip connection led to the best performance.

**Figure 5:**
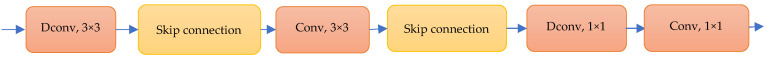
Discovered prediction head structure.

The best-discovered prediction head was composed of two deformable convolution layers with a kernel size of 3 × 3, two skip connections, a convolution layer with a 3 × 3 kernel, and a convolution layer with a 1 × 1 kernel for output. The controller selected only four operations instead of the six allowed operations. The discovered structure provides a good balance between precision and computations. Figure 5 illustrates the structure of the discovered prediction head.

Compared to the prediction head of the original FCOS, the discovered one has fewer parameters and FLOPs while achieving higher performance.

The searched FPN and prediction head were used with a lightweight backbone, the efficientNet, with different variations from b0 to b7. Different configurations for the decoder were proposed to achieve a good trade-off between performance and efficiency. The first has a feature map dimension size of 128, the second has a size of 256, and the third has a size of 128 for the FPN and 256 for the prediction head. The achieved results on the testing set of the proposed dataset with an input image size of 800 pixels are presented in Table 4.

The searched decoder with feature maps with a size of 256 outperformed the original FCOS by 1.7% to 3.9% in AP under different variations of efficientNet. The decoder with feature maps with a size of 128 was very efficient in reducing the number of parameters and the computation overhead, making it suitable for implementation on embedded devices. Notably, the searched decoder with the efficientNet b0 backbone outperformed the original FCOS by 0.9% in AP with fewer FLOPs with a factor of 1/3. The decoder with feature maps with a size of 128 for the FPN and 256 for the prediction head balanced the precision and computation complexity. It is worth noting that the searched decoder surpassed the original one with the same backbone and with fewer parameters and FLOPs.

Moreover, we proved that the proposed NAS method is more efficient than the existing ones. A comparison study is presented in Table 5 to prove the superiority of the proposed NAS method compared to state-of-the-art methods. The proposed NAS can search more architecture per GPU-day than other methods. Compared to DetNAS [30], more than twice the architecture can be searched using the proposed NAS.

**Figure 6:**
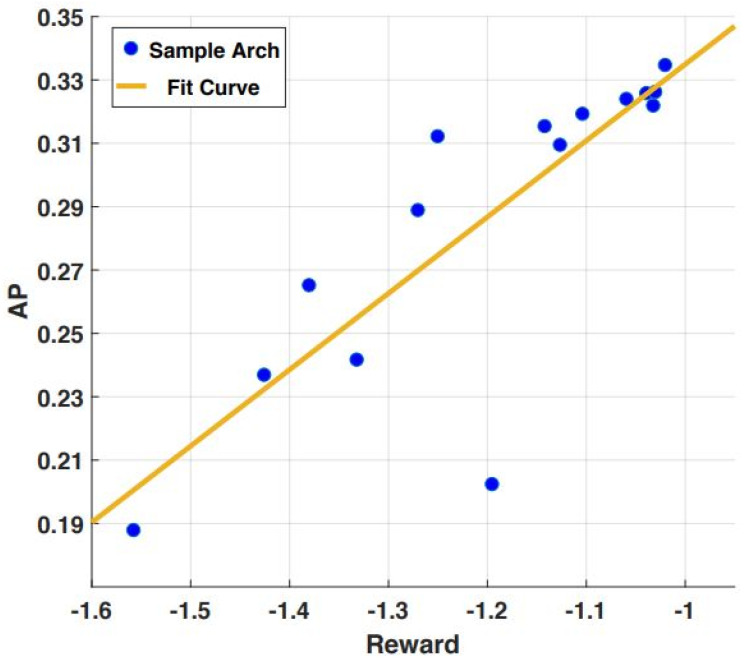
Correlation obtained between achieved AP and search reward.

Furthermore, the reward in the search process was correlated with the proxy dataset and the achieved AP on the proposed dataset for obstacle detection. Figure 6 shows that a high correlation was obtained between the achieved AP and the search reward. Therefore, by investigating the searched architectures, low and high-performance architectures can be observed easily through the reward on the proxy task.

### 4.3. Power Analysis

The proposed model was evaluated on the Xilinx ZCU 102 board. This board comprises a quad-core Arm Cortex-A53, dual-core Cortex-R5F real-time processors, a Mali-400 MP2 graphics processing unit, and programmable logic fabric. In terms of memory, it is equipped with 4 GB of 64-bit memory attached to the processing system and 512 MB of 16-bit memory attached to programmable logic. In addition, the programmable logic used for the acceleration has a high integration capability with 600 K logic cells with 32 MB of on-chip memory and 2520 slices of digital signal processing units (DSP)—the ZCU 102 works under a Peta Linux distribution. The Xilinx Vitis A.I. was used to implement the proposed model. Power consumption was determined by reading the instantaneous power values from the controllers on the hardware platform, accessible through the custom library in the operating system. The overall power consumption resulted from the product of the computation time and the average power value recorded with a period of 0.05 s. The proposed model was quantized to fit the available resources of the ZCU 102 platform. An 8-bit fixed-point representation was used for weights and activations. The model with 128 feature map sizes was evaluated since it is the most suitable for embedded devices. Table 6 presents the achieved results regarding processing speed, mAP after quantization, and power consumption. Due to the quantization of the model, the mAP was slightly decreased. However, the inference speed was good enough to achieve real-time processing, and the power consumption was acceptable.

### 4.4. Quantitative Results

After evaluation and power analysis, the proposed navigation assistance system was evaluated to measure its usability in real-world applications. According to users, six main features can be used for accepting an assistive system by visually impaired people. First, processing speed is a vital feature for system assessment. Respecting real-time processing constraints means detecting obstacles before a certain distance, separating them from the visually impaired. Detecting an obstacle at a distance of less than 1.5 m is preferred. Second, working space can define the performance of the assistive system. Covering indoor and outdoor environments is very important for complete guidance. Finally, the robustness of the proposed system against different challenges, such as occlusion, lighting conditions, and noise, is significant.

Third, the maximum working distance is an important feature. Obtaining more space between the obstacles and the visually impaired person results in safer navigation. Detecting obstacles of different shapes and sizes and detecting moving obstacles are preferred. The portability of the system can be used to assess its performance. A light, economic, and wearable system is more suitable. The friendliness feature defines how easy it is to use the system. An assistive system must be easy to use and not require difficult and long training. A maximum of 19 points is assigned for each feature that presents satisfaction with its conditions. For example, a system that respects real-time constraints will obtain 10 points. A system work for indoor and outdoor spaces will earn 10 points. A system with a working distance of 5 m or more will obtain 10 points. A friendly system will obtain 10 points if it is easy to use and does not require difficult and long training. Table 7 presents a comparison of the proposed system to state-of-the-art assistance systems. In this comparison, only systems based on RGB cameras are considered for fair comparison since the proposed system is based on this camera.

As reported in Table 7, the proposed system outperforms state-of-the-art systems in most assessment features. The findings proved that developing navigation assistance systems based on computer vision and deep learning techniques is very promising and could lead to a trusted assistance system being adopted by the visually impaired community. Unfortunately, designed systems are still far from approaching the human sensing level for environmental understanding. However, coupling more technologies, such as voice recognition and generation, will lead to more powerful assistance systems.

### 4.5. Ablation Study

As mentioned earlier, a wide range of metrics can be used to reward different tasks in NAS, such as AP for object detection and accuracy for image classification. However, it was proved that using AP as a reward for the proxy task does not provide the desired precision for detecting architecture performance in early search stages. Moreover, a deep analysis of the problem revealed that mapping the AP calculation to the decoder reward is complicated. Thus, learning this mapping quickly at the early search stage is impossible. Therefore, we proposed a solution using the negative loss sum on validation data as a reward.

An ablation study was performed through three different experiments to investigate the impact of the search space on the overall performance. First, the FPN was searched using the original prediction head of the FCOS model. Second, the prediction head was searched based on the original FPN of the FCOS model. Third, the FPN and prediction head were searched simultaneously. Table 8 summarizes the achieved results. The results showed that searching the FPN can perform better than searching the prediction head. However, the proposed progressive search method achieved the best performance.

As proved above, deformable convolution layers were the most dominating in the FPN structure and prediction heads due to their ability to adapt to the geometric variation of the target objects. It was identified within each of these findings that including this type of layer is very important for achieving the obtained results. The searched FCOS model achieved better AP than the original FCOS, even when using feature maps with a size of 128.

### 4.6. Use Case: Indoor and Outdoor Navigation

Navigation for visually impaired people is a very complicated task that requires high-performance assistance systems. Navigation must respect social rules, e.g., visually impaired persons must avoid approaching or upsetting individuals who are unwilling to interact with them. In this work, two scenarios have been considered for evaluating the proposed system and the performance of the detection model: first, the problem statement of visually impaired people’s navigation in indoor and outdoor spaces; and second, the use cases for which the proposed system is assessed are specified.

#### 4.6.1. Problem Statement

Most algorithms in the literature have evaluated all obstacles of equivalent significance, including people. This logic does not apply to a visually impaired person, who must be able to travel in the same way that normal people do. It is important to extend the metric and semantic map concepts to cover areas where the visually impaired person may traverse without bothering people and avoiding obstacles that threaten his or her safety.

Supposing two scenarios of navigation, which are indoor navigation and outdoor navigation, for indoor navigation, the proposed system must be able to detect relevant obstacles, such as doors, tables, chairs, and stairs, in addition to other objects, such as light switches, trashcans and fire extinguishers. This ability may facilitate the navigation of the visually impaired person in unfamiliar spaces, such as supermarkets, universities, and hospitals. For outdoor navigation, the system is charged with detecting obstacles that threaten safety, such as vehicles, and other interactive objects, such as pedestrians. To obtain acceptable results, a powerful, intelligent system capable of extracting information from the positions of people and objects, detecting changes in those positions (tracking objects and people), and, of course, knowing the visually impaired person’s physical world at any time is required. It is not a straightforward challenge, and it requires an architecture capable of transferring and processing data in real time.

#### 4.6.2. Use Case Presentation

Two use cases are presented for indoor navigation and outdoor navigation scenarios. Considering that the placement of the camera providing information is on the visually impaired person, a frontal overview is captured and processed for obstacle detection. The proposed navigation assistance system was designed to provide support by providing information about surrounding obstacles. In particular, it communicates with the visually impaired person through audio and vibration.

The first use case scenario describes indoor navigation in which the person is moving in a corridor, and the system provides information about the existence of doors and then warns the user about the existence of stairs. This goal is accomplished by processing the visual data provided by the camera through the proposed object detection model.

The second use case scenario describes indoor navigation in a restaurant: the assistance system provides information about the existing tables and chairs. In addition, the system warns the user of the existence of persons in chairs, so the visually impaired can decide whether the table is available. The third use case scenario concerns moving on a pedestrian path. The system presents the ability to detect moving pedestrians and warn the visually impaired person. The system warns the visually impaired person about interacting with people to avoid passing between them. In addition, the system provides information about trees, traffic signs, and other possible obstacles.

The last use case scenario describes a visually impaired person crossing the street. The proposed assistance system can provide information about the existing vehicles, including cars, buses, motorcycles, bicycles, and trucks. In addition, the proposed system can detect other people crossing the street to help the visually impaired person to decide whether he or she can cross the street. This scenario presents high danger and requires more information to make the final decision. This goal can be accomplished by detecting the traffic light state in some cases.

## 5. Conclusions

Helping visually impaired people to integrate into society is very important and requires high-performance artificial techniques. In this paper, we proposed an obstacle detection system with high performance that is suitable for mobile device implementation. In effect, we proposed to use NAS method to design an object detection model with the desired performance. We proposed the FCOS model as a baseline to optimize search time and computation and searched for the FPN structure prediction head. This process modeled a single-stage anchor-free object detection framework. This specification allowed for accelerating the search time and the investigation of more complex structures more easily. In addition, we proposed the integration of the efficientNet model as a backbone. It is a lightweight CNN model with high performance suitable for implementation on embedded devices. The proposed NAS method was based on an LSTM controller and a reinforcement learning training model. The high-performance decoder for the FCOS model was searched by carefully designing the search space, the proxy task, and the evaluation metric. Extensive experimentation on the proposed dataset, which was composed of the MS Coco dataset and the IDOR dataset, proved the efficiency of the searched decoder integrated with the efficientNet backbone. In future works, we will discuss implementing the obtained model on embedded devices and testing it under real environments.

## Figures and Tables

**Table 1:** Comparison between different object detection models based on different criteria.

Design	Type	Model	Specifications
Manual	Two-stage detectors	RCNN	high computation complexity low detection accuracy slow processing speed
		Faster RCNN	high computation complexity high detection accuracy slow processing speed
	One-stage detectors	Yolo	median computation complexity low detection accuracy fast processing speed
		Yolo v3	median computation complexity good detection accuracy fast processing speed
	Anchor-free detectors	CornerNet	good computation complexity good detection accuracy fast processing speed
		CenterNet	good computation complexity better detection accuracy fast processing speed
Automatic	NAS detectors	DetNAS	good computation complexity good detection accuracy fast processing speed
		NAS-FPN	good computation complexity good detection accuracy fast processing speed

**Table 2:** Operations in the proposed search process.

I.D.	Operation	Kernel Size
0	Depthwise convolution	3 × 3
1	Depthwise convolution with dilation rate 3	3 × 3
2	Depthwise convolution with dilation rate 6	5 × 5
3	Skip connection	--
4	Deformable convolution	3 × 3

**Table 3:** Classes for the obstacle detection system.

Class Name	Class Name	Class Name	Class Name	Class Name
Trash can	Chair	Elevator	Door	Truck
Stairs	Fire hydrant	Table	Stop sign	Heating
Person	Bicycle	Car	moto cycle	Bus
Vase	Couch	Potted plant	Bed	Mirror
Laptop	Microwave	Oven	Desk	T.V.
Street sign	Fire extinguisher	Train	Dining table	Refrigerator

**Table 4:** Comparison between the searched decoder and original decoder for the FCOS model with efficientNet backbone.

Decoder	Size	Backbone	FLOPs (G)	Parameters (M)	AP (%)
Original FCOS	256	EfficientNet b0	103.2	2.4	64.5
		EfficientNet b1	237.7	4.1	70.3
		EfficientNet b2	484.5	6.9	73.6
		EfficientNet b3	856.4	10.4	76.1
		EfficientNet b4	1538.7	19.3	79.3
		EfficientNet b5	3569.2	32.7	81.4
		EfficientNet b6	6291.4	47.2	82.7
		EfficientNet b7	12,946.5	51.4	83.2
FCOS (ours)	256	EfficientNet b0	97.4	2.1	67.3
		EfficientNet b1	202.5	3.8	73.1
		EfficientNet b2	432.8	6.1	76.3
		EfficientNet b3	821.7	9.8	79.7
		EfficientNet b4	1493.5	18.5	82.2
		EfficientNet b5	3108.2	30.4	84.4
		EfficientNet b6	5154.6	44.6	85.8
		EfficientNet b7	10,435.1	49.3	86.3
FCOS (ours)	128/256	EfficientNet b0	92.5	1.8	66.3
		EfficientNet b1	197.4	3.1	72.9
		EfficientNet b2	421.9	7.2	74.5
		EfficientNet b3	810.2	8.4	77.6
		EfficientNet b4	1479.6	16.5	81.8
		EfficientNet b5	3094.3	28.7	83.9
		EfficientNet b6	5140.1	41.4	84.2
		EfficientNet b7	10,427.7	46.2	85.7
FCOS (ours)	128	EfficientNet b0	47.2	1.2	65.6
		EfficientNet b1	100.4	1.9	71.3
		EfficientNet b2	223.6	3.8	73.7
		EfficientNet b3	412.4	4.7	76.2
		EfficientNet b4	802.5	7.9	80.3
		EfficientNet b5	1445.2	13.8	82.9
		EfficientNet b6	2983.8	20.5	83.6
		EfficientNet b7	5201.7	23.7	84.8

**Table 5:** Comparison against state-of-the-art NAS method.

Method	FLOPs (G)	Cost (GPU-Days)	Number of Architectures
NAS-FPN 7 ResNet 50 256 [32]	1125	333× #TPUs	17,000
DetNAS-FPN-Faster [30]	-	44	2200
DetNAS-RetinaNet [30]	-	44	2200
NAS-FCOS-EfficientNet (ours) 256	173.6	26	3000
NAS-FCOS-EfficientNet (ours) 128–256	352.5	26	3000

**Table 6:** Power analysis of the proposed model on the Xilinx ZCU 102 board.

Model	Size	Backbone	F.P.S.	Power (w)	mAP (%)
FCOS (ours)	128	EfficientNet b0	47.2	3.2	63.8
		EfficientNet b1	34.4	3.9	70.1
		EfficientNet b2	32.6	3.8	71.9
		EfficientNet b3	25.4	5.7	74.7
		EfficientNet b4	23.5	5.9	78.6
		EfficientNet b5	21.2	6.8	80.6
		EfficientNet b6	19.8	7.5	81.3
		EfficientNet b7	15.7	7.7	82.9

**Table 7:** Comparison of the proposed system against state-of-the-art assistance systems for visually impaired people.

System	Speed (fps)	Space	Robustness	Working Distance	Friendliness
Smart vision [37]	5	10	3	5	8
Mobile vision [38]	7	5	8	8	9
Mechatronics [39]	10	5	5	10	4
Proposed (ours)	10	10	8	10	8

**Table 8:** Impact of search space on the achieved AP.

Model	Search Space	AP (%)
Original FCOS-EffcientNet b7	-	83.2
FCOS-EffcientNet b7 (ours)	Prediction head	85.1
FCOS-EffcientNet b7 (ours)	FPN	85.6
FCOS-EffcientNet b7 (ours)	FPN + prediction head	86.3

## Data Availability

Data will be made available on request.
